# Variation of Intragenic Tandem Repeat Tract of *tolA* Modulates *Escherichia coli* Stress Tolerance

**DOI:** 10.1371/journal.pone.0047766

**Published:** 2012-10-19

**Authors:** Kai Zhou, Chris W. Michiels, Abram Aertsen

**Affiliations:** Laboratory of Food Microbiology and Leuven Food Science and Nutrition Research Centre (LFoRCe), Department of Microbial and Molecular Systems (M^2^S), Faculty of Bioscience Engineering, KU Leuven, Leuven, Belgium; University of Massachusetts Medical School, United States of America

## Abstract

In recent work we discovered that the intragenic tandem repeat (TR) region of the *tolA* gene is highly variable among different *Escherichia coli* strains. The aim of this study was therefore to investigate the biological function and dynamics of TR variation in *E. coli tolA*. The biological impact of TR variation was examined by comparing the ability of a set of synthetic *tolA* variants with in frame repeat copies varying from 2 to 39 to rescue the altered susceptibility of an *E. coli* Δ*tolA* mutant to deoxycholic acid, sodium dodecyl sulfate, hyperosmolarity, and infection with filamentous bacteriophage. Interestingly, although each of the TolA variants was able to at least partly rescue the Δ*tolA* mutant, the extent was clearly dependent on both the repeat number and the type of stress imposed, indicating the existence of opposing selective forces with regard to the optimal TR copy number. Subsequently, TR dynamics in a clonal population were assayed, and we could demonstrate that TR contractions are RecA dependent and enhanced in a DNA repair deficient *uvrD* background, and can occur at a frequency of 6.9×10^−5^.

## Introduction

DNA sequences harboring tandem repeats (TRs) exist in both prokaryotic and eukaryotic genomes, and are considered to be hypermutable loci in which the TR copy number can increase or decrease as a result of strand-slippage replication or recombination (reviewed in [1]–[3]). The frequency of TR expansions or contractions depends on intrinsic features of the TR tract (such as the length, copy number and sequence conservation of the TR unit) as well as extrinsic environmental conditions [4]–[6]. Obviously, TR rearrangements occurring within promoter or coding regions can affect the transcription and translation of the corresponding genes, or even the functionality of the gene products [7]–[13]. In microorganisms, TR variations are therefore often forwarded as a bet-hedging strategy, from which a population could phenotypically benefit on a short evolutionary time scale [14].

*In silico* analysis of the *E. coli* MG1655 genome readily reveals about 30 genes with an intragenic in frame TR region, in which TR copy number variations thus might affect the functionality of the corresponding protein (unpublished results). However, the effect of TR variation has been studied only in few of these genes. One study showed that in frame expansion of a trimeric (TCT) TR tract from four to five copies in the peroxiredoxin gene *ahpC* converted the enzyme into a disulfide reductase that suppressed loss of fitness in mutants defective in the reduction of protein disulfide bonds [15]. Another study showed that gain or loss of one unit from a three-unit hexameric (CTGGCG) TR tract in the mismatch repair gene *mutL* caused an increased mutation rate. Since the TR region is part of the ATP-binding pocket of MutL, a defective ATPase activity was suggested to have caused the mutator phenotype [16].

The current work focuses on the *tolA* gene, which has a TR region consisting of 13 imperfect repeats of 15 or 18 bp each, and encoding a lysine and alanine rich segment in the TolA membrane protein [17], [18]. As part of the Tol-Pal envelope complex which spans the periplasmic space from the outer membrane to the cytoplasmic membrane and which is important for cell integrity [19], TolA has been implicated in group A colicin uptake [20], [21], filamentous phage infection [22], [23] and detergent tolerance [24]. Notably, the TR region is located within the C-terminal region of domain II of TolA, which comprises a long α-helical domain that connects the cytoplasmic membrane anchor domain I with the periplasmic domain III.

Recent work of our group showed the copy number of *tolA* TR units to vary from 8 to 16 among 234 analyzed *E. coli* isolates [25], but the phenotypical impact of this variation remains unknown. In this study, we therefore aimed to investigate the function and dynamics of TolA TR variation in *E. coli.*

## Materials and Methods

### Bacterial Strains, Plasmids and Growth Conditions

The strains and plasmids used in this study are listed in Table S1. Bacterial strains were grown overnight in well aerated 4-ml cultures of Luria-Bertani (LB) or M9 medium [26] at 37°C, unless mentioned otherwise. For high osmolarity assays, the NaCl concentration of LB was increased to 0.6 M. Antibiotics (Applichem, Darmstadt, Germany) were used at the following concentrations: 100 µg/ml ampicillin (Ap); 25 µg/ml of chloramphenicol (Cm); 10 µg/ml tetracycline (Tc); 50 µg/ml kanamycin (Km); 50 µg/ml streptomycin (St).

### DNA Techniques

Plasmids were isolated using a mini-prep kit (Fermentas, St. Leon-Rot, Germany), and DNA fragments were purified from agarose gels using a gel extraction kit (Fermentas). PCR was performed with DreamTaq polymerase (Fermentas) for examination and Phusion polymerase (Finnzymes, Vantaa, Finland) for cloning and sequencing. Samples for sequencing were prepared with the BigDye terminator V3.1 cycle sequencing kit (ABI, Foster City, CA, U.S.), and sequenced at the division of gene technology (KU Leuven, Belgium).

### Construction of *tolA* Knockouts and Chromosomal TR Variants

A deletion mutant MG1655 Δ*tolA* was constructed using the Datsenko and Wanner method [27]. First, a Δ*tolA*::*kan* fragment containing a kanamycin resistance cassette replacing the *tolA* gene was amplified from strain EVV54 [28] by primer pair *tolA*-Fw (5′-ACTTGAATTCGTAACAGGCGAACAGTTTTT-3′) and *tolA*-Rev (5′-TCGTGGATCCTACCAGAACCCCGTGGCAA-3′), and used to exchange the wild-type *tolA* allele in the chromosome of *E. coli* MG1655, resulting in MG1655 Δ*tolA*::*kan.* Subsequently, MG1655 Δ*tolA* was derived from MG1655 Δ*tolA*::*kan* by flipping out the FRT-flanked *kan* gene.

TR variants of the *tolA* gene were constructed first on a plasmid and subsequently introduced in the chromosome by the following stepwise procedure. First, the wild-type *tolA* allele of MG1655 (i.e. with 13 TR units and further referred to as *tolA* ^13TR^) was PCR-amplified with the *tolA*-Fw and *tolA*-Rev primers, digested with EcoRI and BamHI (Fermentas), and cloned into pTrc99A digested with the same enzymes, to yield pTrc99A-*tolA* ^13TR^. The allele referred to as *tolA* ^†^ harbors two consecutive stop codons in the third TR (TR3), and was constructed by opening pTrc99A-*tolA* ^13TR^ with the back-to-back primer pair *tolA*-pTrc stop Fw (5′-GCTGAAAAGGCTGCAGCTGATTAATAAGCGGCAGCAGAGAAAGC-3′) and *tolA*-pTrc stop Rev (5′-AGCCGCTTTCTTCTCAGCTTCTGCTTTGGCT-3′), of which the former contains the stop codons (underlined). After phosphorylation by PNK T4 kinase (Fermentas), this amplicon was closed again by self-ligation to yield pTrc99A-*tolA* ^†^. To create a *tolA* variant with two repeat units (*tolA* ^2TR^), primer pair 2-repeats-Fw (5′-GCAGAGGCAGATGATATTTTCGGTG-3′) and 2-repeats-Rev (5′-TGCTGCTTTTTCAGCTGCTGCTTTTTCAGCCTTCTCAGCTTCTGC-3′) was used to open pTrc99A-*tolA* ^13TR^ by PCR and at the same time replace the entire 13TR region by two consensus repeat units (underlined), as defined by the Tandem Repeat Finder program [29]. This amplicon was phosphorylated and self-ligated to yield pTrc99A-*tolA* ^2TR^. The *tolA* ^6TR^ and *tolA* ^8TR^ alleles contain 6 and 8 repeats, respectively, were derived from pTrc99A-*tolA* ^†^ by selecting for repeat deletion. Briefly, MG1655 Δ*tolA* containing pTrc99A-*tolA* ^†^ was streaked on LB plates with 0.2% (w/v) deoxycholic acid (DOC, Fisher-scientific, Erembodegem, Belgium). Several DOC tolerant revertants which based on PCR analysis had incurred deletions in the *tolA* ^†^ allele were identified, and a specific pTrc99A-*tolA* ^6TR^ and pTrc99A-*tolA* ^8TR^ construct was retained after confirmation by sequencing. To create a *tolA* ^26TR^ and *tolA* ^39TR^ allele, back-to-back primer pair 2-repeat Fw and 26-repeat Rev (5′-GGCCGCTTTTGCTGCAGCGGCT-3′) was used to open pTrc99A-*tolA* ^13TR^ at the end of the TR region by PCR. Subsequently, this amplicon was ligated with an amplicon of the wild-type *tolA* TR region (containing 13 repeats) obtained with primer pair Pure_TRs Fw (5′-GCTGAGAAGAAAGCGGCTGC-3′) and 26- repeat Rev, to yield pTrc99A-*tolA* ^26TR^ (i.e. single insert) and pTrc99A-*tolA* ^39TR^ (i.e. double insert).

Finally, the *tolA* ^13TR^ wild-type allele and each of the above constructed plasmid-borne *tolA* alleles (except *tolA* ^†^; see below) were amplified with *tolA*-Fw and *tolA*-Rev and individually exchanged with Δ*tolA*::*kan* in the MG1655 Δ*tolA*::*kan* mutant expressing the λ-Red system from pKD46 [27]. Transformants were selected on LB +1% (w/v) sodium dodecyl sulfate (SDS, Applichem, Omaha, U.S.) plates, to which MG1655 Δ*tolA*::*kan* is highly sensitive. As such, MG1655 variants with different chromosomal *tolA* alleles (*tolA* ^2TR^, *tolA* ^6TR^, *tolA* ^8TR^, *tolA* ^13TR^, *tolA* ^26TR^ or *tolA* ^39TR^) were constructed. Loss of the kanamycin resistance cassette and acquisition of the correct allele was corroborated by PCR and sequencing. Please note that the reconstructed MG1655 TolA^13TR^ variant showed no phenotypic differences with the original wild-type MG1655, suggesting that the entire procedure of generating chromosomal *tolA* variants (including a selection on SDS) did not lead to a selection of undesired spontaneous mutants.

The construction of the MG1655 *tolA* ^†^ mutant could not be done by SDS selection because *tolA* ^†^ is a null allele that does not restore SDS tolerance, and was therefore done by using *rpsL* based counterselection [30]. Briefly, the *rpsl*-*neo* cassette was amplified from MG1655 *rpsL150 kdpA4*::*rpsL*-*neo* by primer pair *rpsL*-*neo*_Fw (5′-GCGAACAGTTTTTGGAAACCGAGAGTGTCAAAGGCAACCGGCCTGGTGATGATGGCGGGATCG-3′) and *rpsL*-*neo*_Rev (5′-TGCCTGATGTTGACCGTCCGAACAGTCAACATCGCGATTATCAGAAGAACTCGTCAAGAAGGCG-3′), and then transformed to MG1655 *rpsL150* (St^R^) expressing the λ-Red system from pKD46 to replace *tolA*, generating MG1655 *rpsL150* Δ*tolA*::*rpsL*-*neo* (St^S^). MG1655 *rpsL150 tolA* ^†^ was then obtained by λ-Red based exchange of the Δ*tolA*::*rpsL*-*neo* allele with the *tolA* ^†^ amplicon, and selecting for St resistance.

Strains ZK1, ZK2 and ZK3 were constructed by P1*vir* transduction of antibiotic resistance markers from donor strains *E. coli* TH446 *recA*::*cat* [31], *E. coli* AB1157 *mutS*::Tn10 [32] and *E. coli* JJC40 *uvrD*::Tn5 [33], respectively, into recipient strain MG1655 *rpsL150 tolA* ^†^.

### Extraction of Membrane Proteins and Western Blot

Membrane proteins of *E. coli* MG1655 were extracted as previously described [34]. Briefly, cells from 200 ml stationary phase cultures were harvested by centrifugation for 10 min at 2,900×*g*, resuspended in 10 ml 10 mM Tris-HCl pH 8.0, and lysed by three cycles of freezing and thawing followed by sonication. These samples were centrifuged for 1 hour at 100,000×*g*. The resulting pellet was washed with 10 ml 10 mM Tris-HCl buffer (pH 6.8) supplemented with 1.0 M NaCl and centrifuged again for 1 hour at 100,000×*g*. The pellet was then resuspended in 3 ml of a 10 mM Tris-HCl buffer (pH 6.8) supplemented with 2% Triton X-100, 10 mM MgCl_2_ and 150 mM NaCl to dissolve the membrane proteins. Finally, the remaining cell debris was pelleted for 1 hour at 100.000×*g* and discarded. All steps were performed at 4°C.

Protein samples of about 50 µg as quantified with the Novagen BCA Protein Assay Kit (Merck, Darmstadt, Germany) were boiled for 10 min with loading buffer (50 mM Tris-HCl pH 6.8, 2% SDS, 10% glycerol, 1% β-mercaptoethanol, 12.5 mM EDTA, 0.02% bromophenol blue) and separated on 10% polyacrylamide gel by SDS-PAGE. Western blotting was essentially done as previously described [35], and afterwards the membrane was incubated with a 1∶1000 dilution of the anti-TolA^III^ polyclonal antibodies (PAbs) (a generous gift of Dr. Lloubes, CNRS, France; [36]) in phosphate buffered saline supplement with 0.1% Tween 20 (PBS-T) for 1 hour at 4°C. Subsequently, the membrane was washed with PBS-T for 3×10 min and incubated with horseradish peroxidase (HRP)-conjugated goat anti-rabbit PAbs (1∶30000 dilution) for 1 hour at 4°C. Finally, the blot was washed again in PBS-T before being developed with enhanced chemiluminescence (ECL) substrate solution (Thermo, Rockford, U.S.).

### Determination of Stress Tolerance and Luria-Delbrück Fluctuation Assays

Stationary phase cultures of MG1655 and its derived mutants were serially diluted and plated on LB, LB with 1% DOC (w/v) or 4% SDS (w/v), LB with 0.6 M NaCl (LBS), and LB and LBS adjusted to pH 5.0 with HCl. After overnight incubation at 37°C, colonies were counted and the relative plating efficiency was calculated as (N_d_/N_0_)×100%, with N_d_ = colony-forming units (CFU)/ml on LB with additive(s) and N_0_ =  CFU/ml on LB. MG1655 Δ*tolA* and MG1655 tolA^13TR^ (corresponding to the wild-type) were always included as reference strains.

The Luria-Delbrück fluctuation assay was performed as previously described [37]. Briefly, 22 independent cultures of MG1655 *rpsL150 tolA* ^†^ were grown overnight in LB-broth, after which 10 µl of each culture was separately plated on LB with 1% SDS (referred to as biological replicates). In addition, for one culture, this plating was repeated 22 times (referred to as technical replicates). After overnight incubation, the number of colonies on each plate was counted and the coefficient of variation was subsequently calculated for both the biological and technical replicates as [standard deviation]/[the average number of colonies].

### Tolerance to Infection with Filamentous Phage fd

Phage fd-tet-DOG1 was propagated in *E. coli* TG1 in 2YT medium (16 g Bacto-tryptone, 10 g Bacto-yeast extract, 5 g NaCl in 1L) with 7.5 µg/ml tetracycline at 37°C overnight. After centrifugation (8.000 rpm, 15 min), supernatant containing the phage particles was collected and passed through a 0.2 µm pore-size filter to remove bacteria (Fisher-scientific). Phage titration was done as described previously [26]. Since the primary receptor of phage fd is the tip of the F pilus, the F’ plasmid from *E. coli* XL1-Blue was introduced into each of the MG1655 *tolA* variant strains by conjugation. Hundred µl of a log phase LB culture (OD_600_ ≈ 0.3) of these strains was then mixed with 100 µl fd-tet-DOG1 phage suspension titrated at 100–200 plaque-forming units (PFU), incubated for 30 min at 37°C, mixed with 3 ml LB soft-agar (0.35%) and poured onto an eosin methylene blue (EMB) +1% glycerol agar plate [38]. The plaques, which are dark red on this medium, were counted after overnight growth, and their size was scored. The efficiency of plating phage fd on different mutant backgrounds was expressed relative to that obtained on MG1655 *tolA* ^13TR^, which was arbitrarily set as 100%.

### Statistical Analysis

Plating efficiency experiments were conducted in threefold and results expressed as mean ± standard deviation. Two-tailed unpaired Student’s *t*-test or analysis of variance (ANOVA) followed by Least Significant Different (LSD) post hoc test was used to determine statistical significance of differences between strains, using log-transformed data when necessary.

## Results

### Construction of Different *tolA* TR Variants in *E. coli* MG1655

**Figure 1:**
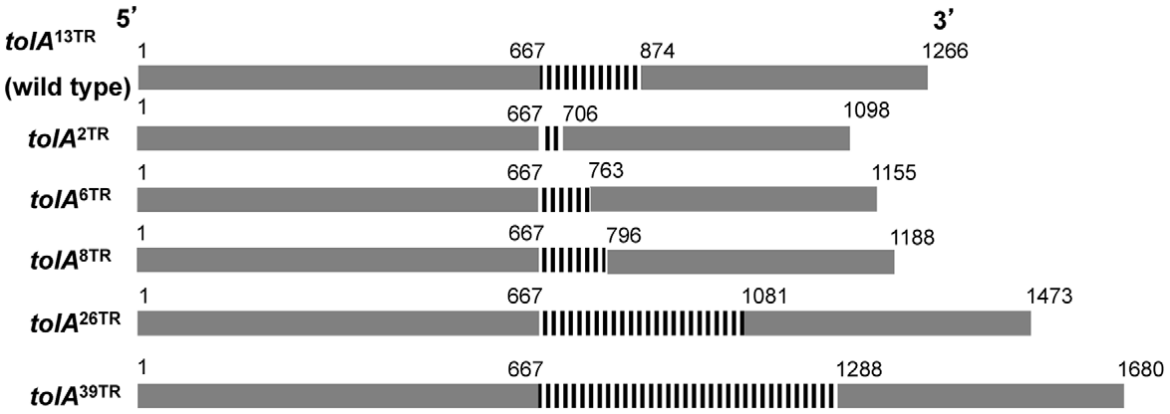
Schematic diagram of *tolA* variants with different TR copy number. All TR variants constructed in this study and introduced in the *E. coli* MG1655 chromosome are shown. Numbers refer to base positions, delineating the start and end points of the *tolA* open reading frame and TR region (vertical black bars).

**Figure 2:**
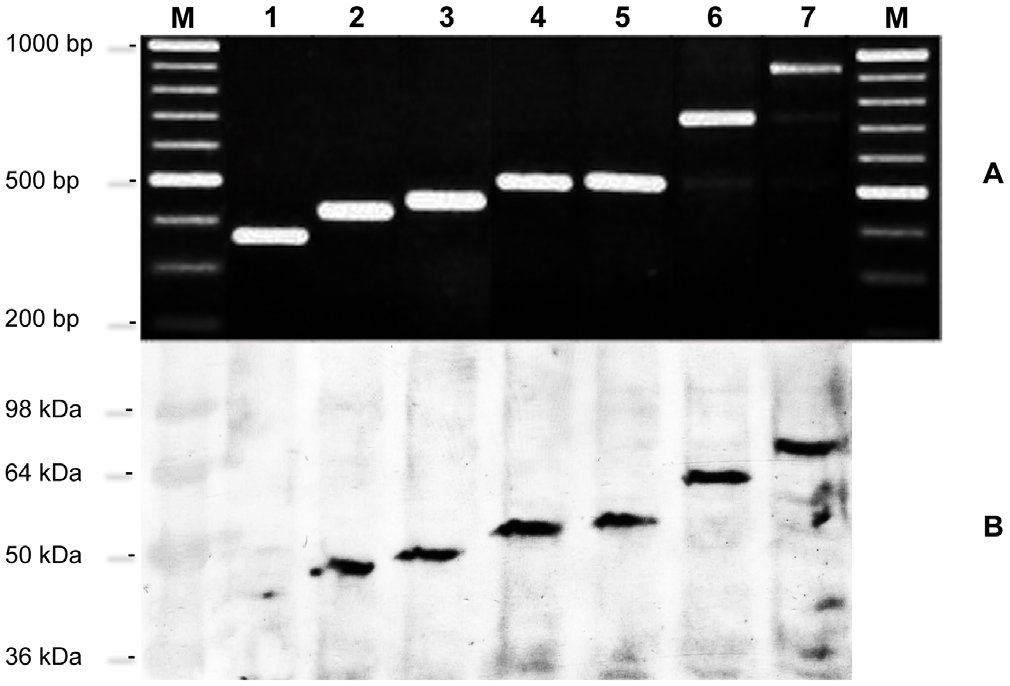
Examination of *tolA* TR variants by PCR and Western blot. (A) PCR analysis of the TR region of *tolA* variants. (B) Western blot of membrane proteins of TolA variants with polyclonal antibody against TolA domain III. Size markers in bp (A) or kDa (B) are shown on the left. M: Marker; Lane 1: TolA^2TR^; Lane 2: TolA^6TR^; Lane 3: TolA^8TR^; Lane 4: wild type (TolA^13TR^); Lane 5: reconstructed wild type (TolA^13TR^); Lane 6: TolA^26TR^; Lane 7: TolA^39TR^.

In order to investigate the function of the TR region of *E. coli* TolA, a set of isogenic MG1655 mutants was constructed differing only in TR copy number in the chromosomal *tolA* locus (Fig. 1). More specifically, TolA variants with 2 (TolA^2TR^), 6 (TolA^6TR^), 8 (TolA^8TR^), 13 (TolA^13TR^, i.e. corresponding to the wild-type TolA protein in MG1655), 26 (TolA^26TR^) or 39 (TolA^39TR^) TR units were generated. While PCR (Fig. 2A) and sequencing (data not shown) confirmed the correct size and sequence of each TR region, Western blotting revealed that TolA^6TR^, TolA^8TR^, TolA^26TR^ and TolA^39TR^ were equally well expressed as the parental TolA^13TR^ (Fig. 2B). In contrast, TolA^2TR^ could hardly be detected by Western blot, indicating that it is either poorly expressed, unstable or unable to react with the applied antibodies that are targeted to domain III of TolA. In fact, since domain II and III have previously been shown to interact [39], the shortened domain II in TolA^2TR^ might impose structural alterations in domain III that preclude its detection with the antibodies used in this study. Whatever the correct explanation, at least some functional TolA^2TR^ is produced in MG1655 *tolA* ^2TRs^ since some of the phenotypes caused by a *tolA* deletion are at least partially reverted in this strain (see further).

### Effect of TolA Repeat Variation on Susceptibility to Infection with Phage fd

Since the TolA protein is structurally involved and essential for entry of filamentous bacteriophages (fd, f1 and M13) in *E. coli* [22], [39], the susceptibility of the Δ*tolA* mutant and the different TolA TR variants to infection with fd phage was compared by determining the phage plating efficiency (Table 1). While the Δ*tolA* mutant displayed resistance to fd infection as expected, all constructed TolA TR copy number variants partially restored phage susceptibility. The fd plating efficiencies on the TolA^2TR^, TolA^6TR^, TolA^8TR^, TolA^26TR^ and TolA^39TR^ variants were not significantly different from each other, but remained significantly lower (28–43%) than those obtained with the control strain expressing the parental TolA^13TR^ (*p*<0.05; ANOVA). Furthermore, the plaque size seemed to be related to plating efficiency, with the TolA^2TR^ variant hosting the smallest plaques. As a control, we also examined the plating efficiency of λ phage on the different TolA TR variants and the Δ*tolA* mutant in the same way, but no significant differences were observed (data not shown). This indicates that the observed differences in fd plating efficiency are probably related to the specific function of TolA in fd infection rather than to an indirect effect on the cell surface properties.

**Table 1:** Plating efficiency of phage fd-tet-DOG1 on *E. coli* MG1655 *tolA^TR^* variants.

Strains	Plating efficiency (%)^1^	Relative plaque size^2^
*ΔtolA*	0*^a^*	–
*2TR*	27.9±5.5*^b^*	+
*6TR*	35.9±7.1*^b^*	++
*8TR*	37.7±3.3*^b^*	++
*13TR*	100±25*^c^*	+++
*26TR*	42.8±3.3*^b^*	++
*39TR*	36.1±3.8*^b^*	++

1 Mean ± standard deviations from three independent experiments, expressed relative to MG1655 TolA^13TR^ (reconstructed wild type; 100%).

2 +++: normal-sized plaques as for MG1655 TolA^13TR^; ++: smaller; +: tiny; -: no plaques detectable.

a,b,c Results with a different letter in superscript are significantly different at 0.05 level by Analysis of Variance (ANOVA).

### Effect of TolA Repeat Variation on Tolerance to DOC and SDS

**Figure 3:**
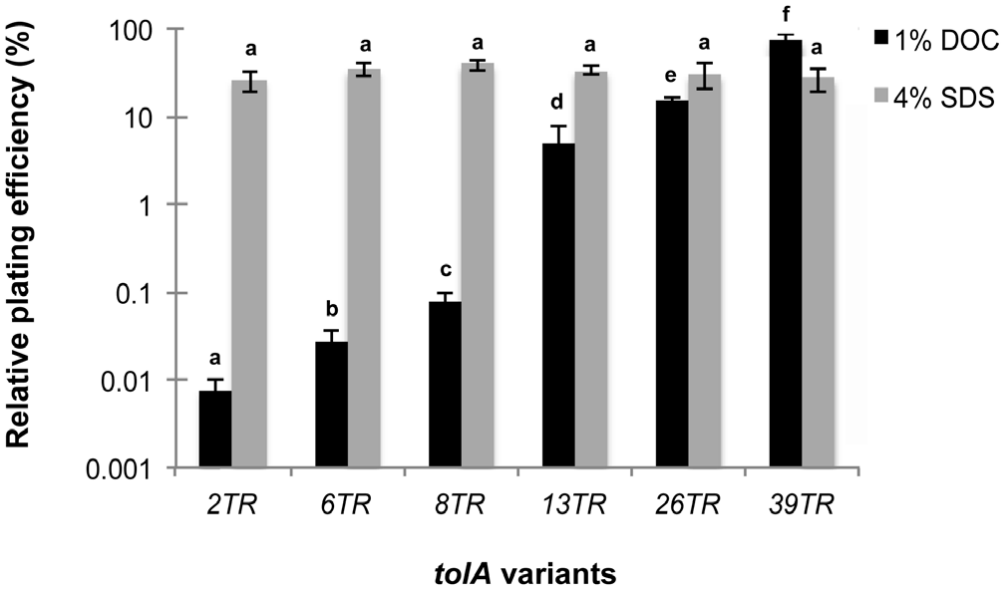
Relative plating efficiencies of MG1655 TolA TR variants on DOC 1% and SDS 4%. The relative plating efficiencies of the Δ*tolA* mutant were <10^−5%^ on both media, and are not shown. 13TR refers to the reconstructed MG1655 TolA^13TR^ strain which is identical to and produced the same results as the wild-type strain MG1655. Error bars represent standard deviations of three independent biological replicates. Bars of the same series marked with a different letter are significantly different at 0.05 level by Analysis of Variance (ANOVA) of log-transformed data followed by LSD post hoc test.

Since TolA is also involved in the maintenance of outer membrane integrity, tolerance to membrane-disrupting agents such as DOC and SDS was determined (Fig. 3). As expected, the Δ*tolA* mutant exhibited hypersensitivity to both DOC (1%) and SDS (4%), with relative plating efficiencies (colony counts on detergent-containing versus detergent-free LB agar) of <10^−5%^ (“<” indicates that no colonies were formed on the detergent plates). The TolA TR variants all showed considerably enhanced DOC tolerance compared to the Δ*tolA* mutant and, interestingly, the degree of tolerance increased with TR copy number (Fig. 3). This correlation was even valid for the variants with increased TR number, which had 3.2-fold (TolA^26TR^) (*p*<0.001; ANOVA) and 15.5-fold (TolA^39TR^) (*p*<0.001; ANOVA) higher plating efficiencies than the reconstructed parental strain (TolA^13TR^).

When colonies of the TolA^2TR^, TolA^6TR^ and TolA^8TR^ variants picked from 1% DOC plates were regrown, they showed comparable plating efficiency with that of TolA^39TR^ on 1% DOC (data not shown), suggesting they had incurred mutations conferring stable DOC resistance. To further analyze these mutants, 150 colonies from 1% DOC were analyzed by PCR to search for possible TR expansion events that could explain their enhanced tolerance. However, no such events were found, which may indicate that the frequency of TR expansions falls below that of TR-independent resistance mechanisms.

The TolA variants also enhanced tolerance to 4% SDS, but in this case tolerance was fully restored to the level of the parental strain by all variants (*p* = 0.458) (Fig. 3). Please note that the construction of the TR variants from the Δ*tolA* strain was based on selection for SDS tolerance, and that this procedure might have allowed the selection of additional SDS-tolerance mutations that could trivially eliminate possible differences between TR variants. To exclude this possibility, the set of TR variants was reconstructed in the absence of any prior SDS selection using *rpsL* based counterselection [30] as described for the construction of MG1655 *tolA* ^†^, and similarly examined for tolerance to 4% SDS and 1% DOC. The corresponding plating efficiencies of this set of mutants were indistinguishable from those shown in Fig. 3 (data not shown), indicating that the observed differences in SDS and DOC resistance can be fully ascribed to the TR variations.

### Influence of TolA Repeat Variation on Sensitivity to High Osmolarity and Low pH

**Figure 4:**
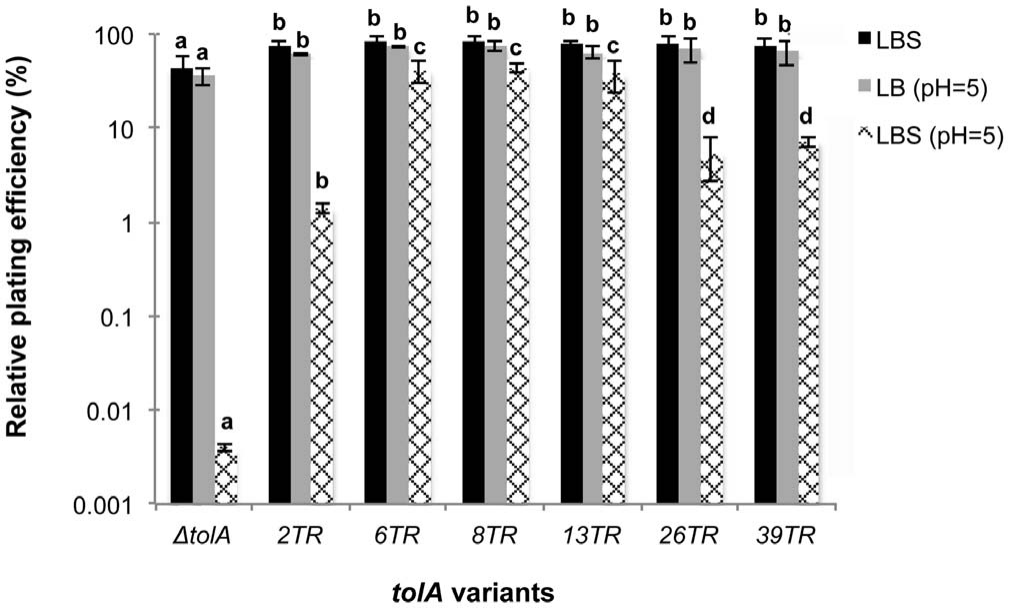
Relative plating efficiencies of MG1655 TolA TR variants on LBS (0.6 M NaCl), LB pH 5 and LBS pH 5 medium. 13TR refers to the reconstructed MG1655 TolA^13TR^ strain which is identical to and produced the same results as the wild-type strain MG1655. Error bars represent standard deviations of three independent biological replicates. Columns of the same series marked with a different letter are significantly different at 0.05 level by Analysis of Variance (ANOVA) of log-transformed data followed by LSD post hoc test.

Another phenotype associated with knock-out of TolA in *E. coli* is reduced growth in LB broth with NaCl at high osmolarity [40]. In agreement with these findings, we observed that the plating efficiency of MG1655 Δ*tolA* on hyperosmotic medium (LBS; 0.6 M NaCl) was around 40%, significantly less than the 80% of the reconstructed wild-type strain expressing TolA^13TR^ (*p*<0.001; ANOVA). All the variant TolA strains had wild-type plating efficiencies (Fig. 4). Interestingly, however, the subsequent combination of hyperosmotic conditions with low pH (LBS pH 5.0) caused more outspoken differences and a differentiation among the TolA^TR^ variants. First of all, the Δ*tolA* strain became hypersensitive, with a plating efficiency of only 0.0004% compared to 38% for the wild-type (*p*<0.001; ANOVA). The strains expressing TolA variants separated into three groups, one with wild-type plating efficiency (TolA^6TR^ and TolA^8TR^), and two with intermediate (1.4–7.3%) plating efficiency (TolA^2TR^, and TolA^26TR^ and TolA^39TR^) (*p*<0.001; ANOVA) (Fig. 4).

### Spontaneous Repeat Variation in TolA

To examine the possible occurrence of variations in repeat copy number within clonal populations, a compromised *tolA* allele (i.e. *tolA* ^†^) harboring two consecutive stop codons in the third repeat was constructed and crossed into MG1655. Just like *tolA* deletion, the *tolA* ^†^ null allele conferred hypersensitivity to DOC and SDS. However, while no colonies with stably reverted SDS or DOC tolerance could be retrieved from the Δ*tolA* mutant, a *tolA* ^†^ mutant population typically yielded such phenotypical revertants at a frequency of 1.9×10^−5^ when plated on 1% SDS and 8×10^−6^ when plated on 0.2% DOC. PCR analysis of the *tolA* locus of 181 such colonies revealed all of them to have incurred contractions in the TR region, with a 5-TR deletion (as evaluated by electrophoretic sizing of the amplified TR region; [25]) being most predominantly retrieved under both DOC (101/104) band SDS (75/77) stress. Furthermore, TolA^†^ revertants obtained on LBS pH 5.0 also typically incurred a 5-TR deletion (17/18).

In addition, a Luria-Delbrück fluctuation assay revealed a much higher coefficient of variation in the mutation frequencies obtained from independent cultures (i.e. 1.4) than that obtained from replicates of a single culture (i.e. 0.2), indicating that TolA^†^ revertants were pre-existing in the population and not induced by selection on SDS.

**Figure 5:**
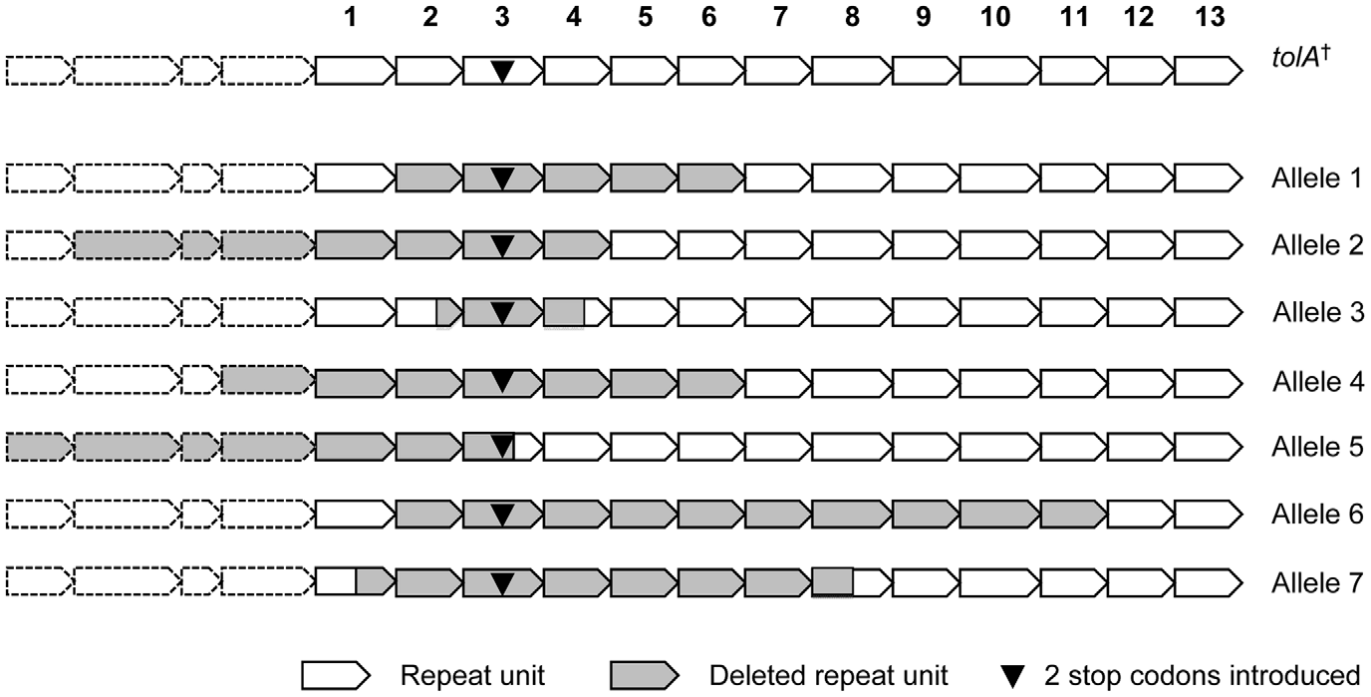
Schematic diagram of *tolA* TR deletions. Different *tolA* TR deletions obtained from MG1655 *tolA*† which carries a nonfunctional *tolA* gene with a TR unit with two stop codons are shown. A total of 39 clones with TR deletions were sequenced, among which seven different TR deletions were found. The 13 TR units that have been recognized in previous studies [17], [18] are numbered. In some cases, the deletions extended in the region upstream of TR1 which contains some less well conserved TR units (dotted lines) (see main text). The differences of unit size were shown in proportion. Allele 1 was found 33 times, both under SDS and DOC selection, and in the *recA, uvrD* and *mutS* backgrounds. All other alleles were found only once. Alleles 2 and 3 were recovered from the strain without additional mutations in recombinational pathways under 0.6 M NaCl pH 5.0 and DOC stress, respectively. Alleles 4 and 5 were recovered from the *recA* background under SDS stress, and alleles 6 and 7 from the *uvrD* background under SDS stress.

Finally, since each TR in *tolA* has a unique sequence, a number of these contracted *tolA* ^†^ loci were sequenced to exactly pinpoint the start and end of the TR deletion (Fig. 5). As expected, the TR unit containing the two stop codons (i.e. TR3) was always deleted, and the deletions were always contiguous and centered around TR3. Moreover, in some of the recovered alleles (e.g. allele 2; Fig. 5) the deletions extended upstream of the TR region as it was previously delineated by us and others. Upon closer inspection, these upstream sequences did also qualify as TRs although they showed a lower degree of sequence conservation, and they could also be identified as TRs when employing different alignment parameter settings (match  = 2, mismatch  = 3, indels  = 5) in Tandem Repeat Finder. Interestingly, a *tolA* gene with exactly the same deletion as allele 2 exists in a natural isolate of *E. coli* (STEC_94C; [25]), confirming that such deletion events can also occur in nature.

### Cellular Functions Involved in TR Contraction in *tolA*

To investigate potential cellular functions affecting TR rearrangements within the *tolA* locus, the impact of key DNA repair (*uvrD* and *mutS*) and recombination (*recA*) proteins on the contraction frequency of the *tolA* ^†^ allele was determined. Since a *recA* mutant is hypersensitive to the DNA damage induced by DOC [41], exposure to 1% SDS was chosen as a selection pressure in these experiments, and the *uvrD*, *mutS* and *recA* mutations themselves did not confer increased sensitivity to this growth condition (data not shown). The TR contraction frequency in the *recA* mutant was only 15% (1.0×10^−5^) of that of the parental strain (6.9×10^−5^) (*p*<0.01; Student’s t-test), indicating these rearrangements to be RecA-dependent. In contrast, an *uvrD* mutation stimulated TR contractions 3.3-fold (2.3×10^−4^) compared to parental levels (*p*<0.01; Student’s t-test). Finally, *mutS* (3.8×10^−5^) had no significant effect on repeat stability in *tolA* ^†^ (*p*>0.05; Student’s t-test).

As in the wild-type background, a 5-TR deletion also was predominantly retrieved under both DOC (64/66) and SDS (37/40) stress in the various DNA repair and recombination deficient backgrounds. Furthermore, upon sequencing, some novel rearranged *tolA* alleles were recovered from *recA* and *uvrD* mutants under SDS stress, some of which also involved the upstream sequences of the originally assigned TR region (Fig. 5).

## Discussion

In this study, we investigated the possible biological function and dynamics of TR variation in the *E. coli tolA* gene. Comparison of a constructed set of isogenic mutants varying only in the copy number of in frame TR units in the *tolA* gene, revealed that each of these TolA TR variants was able to rescue the aberrant phenotypes incurred by a Δ*tolA* mutant in response to various biological and chemical stresses, although the extent of this complementation was dependent on both the TR copy number and the type of stress imposed.

The most outspoken TR-dependent phenotype was DOC tolerance, for which plating efficiencies increased with an increasing number of TR units from TolA^2TR^ to TolA^39TR^ over a range of four orders of magnitude. DOC is the major component of bile salts, which constitute a major stress factor for *E. coli* and other bacteria in the mammalian gut. In fact, bile salts have recently been recognized as an important evolutionary selection force, contributing to the diversification of enteric species such as *E. coli* and *Salmonella enterica* [42]. As a result, a number of bile resistance mechanisms have already been identified and documented, mainly involving efflux pumps (AcrAB and EmrAB), outer membrane proteins (OmpF and OmpC), SOS response, and two-component systems (i.e. PhoPQ) ([43]; also reviewed in [41], [44]). Nevertheless, this study is the first to demonstrate that variation of TolA TR copy numbers can modulate DOC tolerance in *E. coli*.

In contrast to DOC sensitivity, all TolA TR variants complemented sensitivity to SDS and hyperosmolarity equally well and up to wild-type level. However, when hyperosmolarity was combined with low pH, the TolA^6TR^, TolA^8TR^, and TolA^13TR^ strains outperformed the other variants carrying either lower or higher TR copy numbers. Although the exact molecular mechanisms behind such differences remains to be elucidated, these findings underscore the intricate phenotypical changes brought about by TolA TR variation.

Finally, all TolA variants were significantly less susceptible to filamentous phage fd than the strain expressing wild-type TolA (i.e. TolA^13TR^). Since entry of fd requires specific interaction of the phage minor coat gene 3 protein (G3p) with domain III of the TolA protein, the reduced fd sensitivity of the TR variants may be due to an allosteric effect of the TR-dependent variations in the length of domain II on the proper presentation of domain III. This hypothesis is further supported by the fact that domain II and III have previously been shown to physically interact [39].

From an ecological perspective, the different stresses mentioned above represent a number of opposing selective forces with regard to the optimal TR copy number in the *tolA* gene. Exposure to DOC, for example, is anticipated to be a strong selective force for increasing TR copy numbers, which would in turn attenuate tolerance to high osmolarity combined with low pH. We previously reported *tolA* alleles with TR copy numbers varying from 8 to 16 among 234 natural *E. coli* strains, with *tolA* ^13TR^ occurring in 66% of the strains, although the frequency distribution seemed to be different for some pathogens [25]. Our current findings suggest that 13 TRs may indeed provide an optimal tolerance to the different chemical stresses investigated in this study (DOC, SDS, high NaCl concentration, and high NaCl concentration at low pH).

The *tolA* ^†^ allele carrying two stop codons in one of the TR units that was constructed in this work allowed us to demonstrate that TolA TR variations occur in a clonal wild-type population at a frequency of at least 6.9×10^−5^, thereby proving that TolA TRs can dynamically change on short evolutionary time scales. Moreover, these TR rearrangements were supported by RecA but suppressed by UvrD. Since RecA and UvrD are known to support and suppress homologous recombination [33], [45], respectively, these findings suggest that recombination is the primary mechanism affecting instability of the *tolA* TRs in *E. coli*. In contrast, although MutS has been shown to stimulate the rearrangement frequency of dimeric TRs [46], knocking-out *mutS* had no effect on contractions of the 15-mer TRs in our experiments. This observation is likely explained by the fact that DNA mismatch repair mainly targets nucleotide mismatches and insertion/deletion bulges of only 1–4 bp in length [47]. A similar conclusion was drawn from a previous study, which showed that *mutS* deficiency did not affect the mutation frequency at any of the 28 variable-number tandem repeats (VNTRs) with TR unit sizes >5 bp in *E. coli* O157:H7 [5].

Finally, it is noteworthy that rearrangement of the *tolA* ^†^ allele typically resulted in 5-TR deletions. Moreover, neither the 12^th^ nor 13^th^ TR was ever shown to take part in contraction events. Possibly, these two repeats are essential for TolA function, and it was indeed suggested in a recent study that 31 residues at the C-terminal end of domain II of TolA (including the 12^th^ and 13^th^ TR) are required for binding the tetratricopeptide repeat domain of YbgF in the Tol-Pal complex, thereby controlling oligomeric state of YbgF [48].

In conclusion, this study demonstrates the pleiotropic phenotypic effects of TR copy number variations in the *E. coli tolA* gene, thereby revealing some possible selective forces able to drive TR rearrangements. Moreover, recombination-dependent TR rearrangements in *tolA* could be detected in clonal populations, further supporting a role of TR regions as hypermutable contingency loci that allow rapid and flexible adaptation to complex environmental conditions.

## Supporting Information

Table S1 **Strains and plasmids used in this study.**
(DOCX)
