# Supplementary material for: Variation of Intragenic Tandem Repeat Tract of tolA Modulates Escherichia coli Stress Tolerance
Source: PLoS One. 2012 Oct 19;7(10):e47766. doi: 10.1371/journal.pone.0047766 (PMC3477136; doi:10.1371/journal.pone.0047766)
Supplement: Table S1 — Strains and plasmids used in this study. (DOCX) [file pone.0047766.s001.docx]

Table S1 Strains and plasmids used in this study

| Strain or plasmid | Relevant features | Source or reference |
| --- | --- | --- |
| *E. coli* strains |  |  |
| MG1655 | K12, F^-^ λ^-^ *ilvG*- *rfb*-50 *rph*-1 | [1] |
| EVV54 | W3110 *Δ*(*argF*-*lac*) U169 λRS88 (*degP*-*lacZ*) *ΔtolA*::*kan*, Km^R^ | [2] |
| MG1655 *ΔtolA*::*kan* | *tolA* gene replaced by *kan* cassette, Km^R^ | This study |
| MG1655 *ΔtolA* | deletion of *tolA* gene | This study |
| MG1655 *rpsL150* | St^R^ | [3] |
| MG1655 *rpsL150 kdpA4*::*rpsL-neo* | St^S^, Km^R^ | [3] |
| MG1655 *rpsL150* Δ*tolA*::*rpsL-neo* | St^S^, Km^R^ | This study |
| MG1655 *rpsL150* *tolA*^†^ | *tolA* containing two stop codons, St^R^ | This study |
| MG1655 *tolA*^2TR^ | *tolA* variant with 2 consensus TRs | This study |
| MG1655 *tolA*^6TR^ | *tolA* variant with 6 TRs | This study |
| MG1655 *tolA*^8TR^ | *tolA* variant with 8 TRs | This study |
| MG1655 *tolA*^13TR^ | *tolA* variant with 13 TRs (same as MG1655) | This study |
| MG1655 *tolA*^26TR^ | *tolA* variant with 26 TRs | This study |
| MG1655 *tolA*^39TR^ | *tolA* variant with 39 TRs | This study |
| MG1655 *ΔtolA* F' | susceptible to fd phage, Tc^R^ | This study |
| MG1655 *tolA*^2TR^ F' | susceptible to fd phage, Tc^R^ | This study |
| MG1655 *tolA*^6TR^ F' | susceptible to fd phage, Tc^R^ | This study |
| MG1655 *tolA*^8TR^ F' | susceptible to fd phage, Tc^R^ | This study |
| MG1655 *tolA*^13TR^ F' | susceptible to fd phage, Tc^R^ | This study |
| MG1655 *tolA*^26TR^ F' | susceptible to fd phage, Tc^R^ | This study |
| MG1655 *tolA*^39TR^ F' | susceptible to fd phage, Tc^R^ | This study |
| DH5α | F^-^ *endA*1 *glnV*44 *thi*-1 *recA*1 *relA*1 *gyrA*96 *deoR nupG* Φ80d*lacZ Δ*M15 *Δ*(*lacZYA-argF*)U169, *hsdR*17(r_K_^-^ m_K_^+^), λ^–^ | [4] |
| TG1 | K-12, F' [*traD*36 *proAB^+^ lacI^q^ lacZΔM*15],  *supE thi-1 Δ*(*lac-proAB*) *Δ*(*mcrB-hsdSM*)5*,* (*r_K_^-^m_K_^-^*), harboring Fd-tet-DOG1 phage, Tc^R^ | Patrice Soumillion, Université Catholique de Louvain, Belgium |
| XL1-Blue | *endA*1 *gyrA*96(nal^R^) *thi-1* *recA*1 *relA*1 *lac glnV*44 F'[::Tn10 *proAB*^+^ l*acI^q^* *Δ*(*lacZ*)M15] *hsdR*17(r_K_^-^ m_K_^+^) | CMPG, K.U.Leuven |
| TH446 *recA*::*cat* | MG1655, *recA*::*cat,* Cm^R^ | [5] |
| YG2273 | AB1157, *mutS*::Tn10, Tc^R^ | [6] |
| JJC212 | AB1157 *hsdR*, *uvrD*::Tn5, Km^R^ | [7] |
| ZK1 | MG1655 *rpsL150 tolA*^†^, *recA*::*cat*, Cm^R^, St^R^ | This study |
| ZK2 | MG1655 *rpsL150 tolA*^†^, *mutS*::Tn10, Tc^R^, St^R^ | This study |
| ZK3 | MG1655 *rpsL150 tolA*^†^*, uvrD*::Tn5, Km^R^, St^R^ | This study |
| Plasmids |  |  |
| pTrc99A | Cloning vector carrying IPTG-inducible *trc* promoter | [8] |
| pTrc99A-*tolA*^†^ | pTrc99A carrying *tolA* with 2 stop codons | This study |
| pTrc99A-*tolA*^2TR^ | pTrc99A carrying *tolA* with 2 consensus TRs | This study |
| pTrc99A-*tolA*^6TR^ | pTrc99A carrying *tolA* with 6 TRs | This study |
| pTrc99A-*tolA*^8TR^ | pTrc99A carrying *tolA* with 8 TRs | This study |
| pTrc99A-*tolA*^13TR^ | pTrc99A carrying *tolA* with 13 TRs | This study |
| pTrc99A-*tolA*^26TR^ | pTrc99A carrying *tolA* with 26 TRs | This study |
| pTrc99A-*tolA*^39TR^ | pTrc99A carrying *tolA* with 39 TRs | This study |
| pKD46 | encoding arabinose-inducible λ-Red recombinase | [9] |

**References**

1. Guyer MS, Reed RR, Steitz JA, Low KB (1981) Identification of a sex-factor-affinity site in *E. coli* as gamma delta. Cold Spring Harb Symp Quant Biol 45: 135-140.
2. Vinés ED, Marolda CL, Balachandran A, Valvano MA (2005) Defective O-antigen polymerization in *tolA* and *pal* mutants of *Escherichia coli* in response to extracytoplasmic stress. J Bacteriol 187: 3359-3368.
3. Heermann R, Zeppenfeld T, Jung K (2008) Simple generation of site-directed point mutations in the *Escherichia coli* chromosome using Red(R)/ET(R) Recombination. Microb Cell Fact 7: 14.
4. Grant SG, Jessee J, Bloom FR, Hanahan D (1990) Differential plasmid rescue from transgenic mouse DNAs into *Escherichia coli* methylation-restriction mutants. Proc Natl Acad Sci U S A 87: 4645-4649.
5. Hill TM, Sharma B, Valjavec-Gratian M, Smith J (1997) *sfi*-independent filamentation in *Escherichia coli* Is *lexA* dependent and requires DNA damage for induction. J Bacteriol 179: 1931-1939.
6. Wagner J, Nohmi T (2000) *Escherichia coli* DNA polymerase IV mutator activity: genetic requirements and mutational specificity. J Bacteriol 182: 4587-4595.
7. Bierne H, Seigneur M, Ehrlich SD, Michel B (1997) *uvrD* mutations enhance tandem repeat deletion in the *Escherichia coli* chromosome via SOS induction of the RecF recombination pathway. Mol Microbiol 26: 557-567.
8. Amann E, Ochs B,  Abel KJ (1988) Tightly regulated *tac* promoter vectors useful for the expression of unfused and fused proteins in *Escherichia coli*. Gene 69: 301-315.
9. Datsenko KA, Wanner BL (2000) One-step inactivation of chromosomal genes in *Escherichia coli* K-12 using PCR products. Proc Natl Acad Sci U S A 97: 6640-6645.
